# Development and initial validation of an online engagement metric using virtual patients

**DOI:** 10.1186/s12909-018-1322-z

**Published:** 2018-09-17

**Authors:** Norman B. Berman, Anthony R. Artino

**Affiliations:** 1 Dartmouth Geisel School of Medicine, One Rope Ferry Road, Hanover, NH 03756 USA; 2 Medicine, Uniformed Services University, 4301 Jones Bridge Road, Bethesda, MD 20814 USA; 3 One Medical Center Drive, Bethesda, NH 03756 Lebanon

## Abstract

**Background:**

Considerable evidence in the learning sciences demonstrates the importance of engagement in online learning environments. The purpose of this work was to demonstrate feasibility and to develop and collect initial validity evidence for a computer-generated dynamic engagement score based on student interactions in an online learning environment, in this case virtual patients used for clinical education.

**Methods:**

The study involved third-year medical students using virtual patient cases as a standard component of their educational program at more than 125 accredited US and Canadian medical schools. The engagement metric algorithm included four equally weighted components of student interactions with the virtual patient. We developed a self-report measure of motivational, emotional, and cognitive engagement and conducted confirmatory factor analysis to assess the validity of the survey responses. We gathered additional validity evidence through educator reviews, factor analysis of the metric, and correlations between student use of the engagement metric and self-report measures of learner engagement.

**Results:**

Confirmatory factor analysis substantiated the hypothesized four-factor structure of the survey scales. Educator reviews demonstrated a high level of agreement with content and scoring cut-points (mean Pearson correlation 0.98; mean intra-class correlation 0.98). Confirmatory factor analysis yielded an acceptable fit to a one-factor model of the engagement score components. Correlations of the engagement score with self-report measures were statistically significant and in the predicted directions.

**Conclusions:**

We present initial validity evidence for a dynamic online engagement metric based on student interactions in a virtual patient case. We discuss potential uses of such an engagement metric including better understanding of student interactions with online learning, improving engagement through instructional design and interpretation of learning analytics output.

## Background

Considerable evidence in education and the learning sciences demonstrates a robust, positive association between engagement and academic achievement [1]. This relationship is especially apparent (and essential) in online learning environments where a live instructor is absent. Sinatra emphasizes the importance of “grain size” when considering a measurement of engagement [2]. Engagement in education has often referred to macro-level behaviors such as attendance and participation in school activities. Micro-level measures of engagement also exist, such as eye movement and heart rate. In online learning environments, data to assess engagement may be available at both the micro and macro levels.

Engagement is typically conceptualized as having three domains – behavioral (conduct, effort, participation), affective (interest, attitude, emotion) and cognitive (self-regulation, cognitive investment) [1]. Behavioral engagement as it relates to online learning can be seen in the actions a learner takes that reflect involvement in learning and academic tasks, such as time on task and answer accuracy, which often mirror effort, persistence and attention [3]. Affective engagement in online learning involves both positive and negative reactions to the learning environment and content, and is closely linked to motivation [4]. Cognitive engagement has been difficult to operationalize as a single construct as it intersects with behavioral and affective engagement. Cognitive engagement can be defined as “the extent to which students’ are willing and able to take on the learning task at hand” [5], or alternatively “integration and utilization of students’ motivations and strategies in the course of their learning” [6].

Virtual patients (VP), one form of online learning, are used increasingly in medical education [7]. Using a framework described by Kononowicz et al. [8], VPs can be defined rather simply as multimedia screen-based interactive patient scenarios. Despite the now substantial level of use, little is known about the features of VP design or factors of VP use that will best promote learning [9]. Effective learning in the autonomous online environment typical of VP use requires learner motivation and self-regulation [10]. However, the learning environment typical of VP use does not inherently foster learner engagement [11] and therefore may not ideally support high-quality learning. Educators choosing to use VPs in their courses are often sending their learners into a “black box”, where they are blind to the ways in which learners are interacting (or not) with the instructional materials. Experience with VP use suggests that students will not complete many VPs unless required [12] and that requiring VP use is not the best integration strategy [13]. This suggests a need for a way to assess learner engagement with VPs.

Survey instruments exist for measuring cognitive engagement in problem-based learning classrooms [5], and a survey instrument exists to evaluate the related concepts of task value and self-efficacy in online learning [10]. To our knowledge, there are no instruments (survey or otherwise) for measuring engagement in the setting of VP use in medical education.

The overarching objective of this work was to demonstrate feasibility and to develop and collect initial validity evidence for a computer-generated dynamic engagement score based on student interactions in an online learning environment, in this case virtual patients used for clinical education. Our primary research question was the following: Do student actions while completing an online virtual patient case reflect their engagement? For the purpose of this study, we operationalize engagement as the integration and utilization of students’ motivations and learning strategies in the course of their learning.

## Methods

### Participants and setting

The study involved third-year medical students at more than 125 accredited US and Canadian medical schools at which VP cases were a standard component of their educational program in the Pediatrics, Internal Medicine and Family Medicine clerkships. The VPs used were from a centralized VP bank accessible from a US based non-profit, and were designed to meet nationally accepted curricular guidelines. Each course included from 30 to 35 cases, with cases taking students, on average 30–45 min to complete. Use of these VPs was defined by the respective medical schools, with some requiring completion of a specified number of VPs, while in other schools use was optional. The total population of student users was more than 15,000 over an academic year. A dynamically generated engagement meter with red, yellow or green circles corresponding to low, moderate or good engagement, was displayed to students as a routine aspect of VP case use beginning in July 2013. The color displayed on each page depended on student actions on preceding pages, as determined by the scoring algorithm. Surveys were included within cases in September 2014.

### Measures

#### Engagement metric

We developed our engagement metric based on review and analysis of a widely used VP software package, CASUS [14]. We considered all student actions within a VP which are measureable and may reflect engagement for inclusion in the engagement metric. The engagement metric algorithm we developed included four equally weighted components of student interactions with the case: time on page, multiple-choice question answer accuracy, use of a clinical reasoning tool, and scoring of students’ written summary statements based on the VP encounter.

- Time in seconds spent on each page in a VP encounter was scored as 0 or 1, based on actual time above or below an empirically derived 20 s cut-point; this component score was calculated as a percent of the possible total (0 to 100%), which varied by number of pages in the case.
- Multiple-choice questions (MCQ) were embedded throughout VP cases, but were not included on every case page. MCQ accuracy was scored cumulatively as the learner progressed through the case and completed more questions; this component score was calculated as a percent (0-100%) of the possible total, which varied by number of questions in the case.
- The VP software included a clinical reasoning toolbar that allowed free-text entry of key findings and differential diagnosis as the case progresses. Students were able to use the toolbar at any point in the case. The VP software counted entry of key findings, and additions, deletions and changes in the rank order of the differential diagnosis, with each action counted equally and scored cumulatively. Based on review of student log data, the total score achieved was calculated as a percentage (0 to 100%) of an empirically derived maximum of 12 actions (no additional credit given for score > 12).
- Students were given the opportunity to write one summary statement per case based on the findings in the VP encounter. Written summary statements were analyzed by machine learning software [15], which was trained to correlate student text input with a specific case. Training was based on 500–1000 writing samples from each VP case. Credit was given if the machine learning software correctly predicted the case number with a certainty greater than 50%. Summary statement score match was included in the algorithm as a binary 0 or 1 score.

The final engagement score was calculated as a mean of the four sub-scores. The scoring algorithm cut-points for determining low, moderate or good engagement were empirically derived after reviewing randomly selected log data from 20 students. Scores < 0.3 were considered low; 0.3 to 0.5 were considered moderate; and > 0.5 were considered good.

#### Engagement survey

We assessed validity evidence for relations of the engagement metric to other variables by calculating the correlations between the engagement score and four self-report measures (survey scales) of motivational, emotional, and cognitive engagement: *task value* (motivational engagement); *boredom* (emotional engagement); *elaboration* and *engagement* (cognitive engagement). All four scales were adapted from previously published instruments, as described below, and each survey item employed a five-point, Likert-type response scale: 1 = not at all true for me; 2 = slightly true for me; 3 = moderately true for me; 4 = mostly true for me; 5 = completely true for me.

##### Motivational engagement

We measured students’ self-reported motivational engagement using a five-item *task value* scale adapted from Artino and McCoach [16]. The scale assessed students’ judgments of how interesting, important, and useful the VP case activity was to them. Several minor wording changes were made to the task value items; these changes addressed the differences between the original survey context and the VP context studied in the present investigation. Sample items include “Overall, I was very interested in the content of the cases” and “The cases provided me with a great deal of practical information.”

##### Emotional engagement

We measured students’ self-reported emotional engagement using a four-item *boredom* scale adapted from the Achievement Emotions Questionnaire (AEQ) [17]. The scale assessed students’ case-related boredom. Once again, changes were made to the original scale items to reflect the VP context studied here. Sample items include “I was bored while completing the cases” and “My mind tended to wander while completing the cases.” It is worth noting that similar versions of this modified AEQ have been employed in previously published research [11, 18].

##### Cognitive engagement

We measured students’ self-reported cognitive engagement using two scales: a four-item *elaboration* scale adapted from the Motivated Strategies for Learning Questionnaire [19] and a three-item *engagement* scale adapted from Rotgans and Schmidt [5]. The elaboration scale assessed students’ use of cognitive strategies while completing the cases (e.g., paraphrasing and summarizing case content; linking new content to previously learned information), and the engagement scale assessed students’ judgments of “situational cognitive engagement” [5]. Once again, changes were made to the original scale items to reflect the VP context studied here. Sample items for elaboration include “I tried to relate what I was learning during the cases to what I already know” and “I tried to connect what I was learning in the cases with my own experiences.” Sample items for engagement include “I was engaged with the topics of the cases” and “I put in a lot of effort while completing the cases.”

### Procedures

#### Engagement metric validation

We used Messick’s framework [20] to guide our collection of validity evidence for the engagement metric. To assess content validity, we surveyed six medical educators from six different institutions about the engagement metric components. These educators were shown log data from students’ completion of 10 sample cases and were asked “Do you feel that you are able to assess student engagement by looking at these data?” Further, to assess an aspect of consequential validity, we considered the appropriateness of the chosen scoring cut-points [21, 22]. Evidence supporting the validity of the scoring cut-points was collected from the same 6 medical educators, who reviewed log data from students’ completion of 10 sample cases and rated student engagement as low, moderate or good. (See Table 1 for examples.) We also evaluated the distribution of low, moderate and high engagement scores after implementation of the metric.

**Table 1:** Examples of data demonstrating low, moderate and good engagement

Page	Case A - Low				Case B - Moderate				Case C - Good			
	Time	MCQ	CR	Summary	Time	MCQ	CR	Summary	Time	MCQ	CR	Summary
1	7				11				393	100		
2	10				13	0			193		1	
3	27				17				317	71	11	
4	10				14	43			281		2	
5	14				43				604	43	1	
6	4				26	0			147	100		
7	73	0			28				873			
8	23			0	10				124	0	2	
9	19	27			14	0			110	100		
10	4				9	20			122		1	
11	18				11				41			
12	11				6				85			
13	10				8				136	67	1	
14	12				6	0			95			
15	6				21				56	100		
16	28	60			7	0	2		168			
17	8	0			22				58	75		
18	3				10	0			121		1	1
19	11	20			79				197			
20	9	33			17				294	14		
21	4				56	43			407			
22	14	0			8			1	223	100		
23	7	100			5	0			768			
24	9				15				46			
25	4				15				522			
26	1								23			
27	9								8			
28									609			
Comp	0.15	0.3	0	0	0.28	0.10	0.17	1	0.96	0.7	1	1
Score	0.11				0.39				0.92			

Table 1 demonstrates examples of user interaction data and scoring from 3 different cases, each with a different level of engagement. Final engagement score < 0.3 = low; 0.3 to 0.5 = moderate; > 0.5 = good. Time is in seconds per page. MCQ = multiple choice question; data is shown only for pages that include an MCQ. CR = clinical reasoning; data is shown only for pages in which a student action occurred. Summary = summary statement; data is shown only for the page that included a summary statement question.

Page = page number. Comp = component score. Score = final engagement score.

To assess internal structure, we conducted a confirmatory factor analysis (CFA) of the four components of the engagement score using the case use data from 1400 students who were randomly selected from the larger set of student case use data during 2014 and 2015. We analyzed only use of cases that incorporated a summary statement, for a total of 37 cases. We also considered the test-retest reliability of the scoring system. The computer-based collection and scoring of each component of the engagement metric, and of the final score, also provides evidence of response process validity.

To assess validity evidence for relationship with other variables, we examined the relationship between the engagement metric and the various engagement survey scales. A draft student survey was pilot tested on a VP case page at the conclusion of the cases. Exploratory factor analysis suggested a four-factor structure of the four survey scales. Each scale (motivational, emotional, and two cognitive engagement scales) was administered independently, so that the order in which students would see each scale was quasi-random.

#### Engagement survey administration

The final 16-item survey, which included the motivational, emotional and two cognitive sub-scales, was administered as a single online survey at the end of individual cases. Survey responses were linked by software to individual case sessions.

Survey responses were de-identified by project staff and were not shared with individuals involved in grading decisions at the students’ school. Participation in the survey was voluntary, and no incentives were given for survey completion. The Dartmouth College Committee for the Protection of Human Subjects exempted the study from further review.

### Statistical analysis

We assessed rater agreement with the empirically derived scoring cut-points using Pearson correlation. We assessed inter-rater reliability using intra-class correlation for these ratings.

Prior to analysis of the survey instrument, we screened the data for accuracy and missing values and checked each variable pattern for normality. We conducted confirmatory factor analysis (CFA) on the draft student survey, and then conducted a CFA on the full survey responses to assess the convergent and discriminant validity of the 16 survey items that comprised the four scales. We used maximum likelihood estimation to estimate the parameters, and we inspected several goodness-of-fit statistics to evaluate model fit [23]. Next, we subjected each of the four scales to an internal consistency reliability analysis (Cronbach’s alpha) and computed a mean score for the items associated with a particular scale (i.e., the four variables were un-weighted composite scores). Third, we calculated descriptive statistics and conducted a correlation analysis to explore the associations among the survey variables and the case-generated engagement score for all participants. Students completing more than one survey were treated independently. Both CFAs were completed using M*plus*, Ver. 7.4, and all other analyses were conducted with IBM SPSS Statistics, Ver. 22.0 (IBM Corporation, New York, NY).

## Results

### (participants)

1807 surveys were completed by 1254 students, at 149 institutions. Surveys were completed for cases in Internal Medicine, Pediatrics and Family Medicine. All students completing more than one survey did so on different cases. Range of surveys completed per student is from 1 to 12, mean 1.44. Surveys completed per case ranged from 2 to 103, mean 18.3.

### (measures)

#### Survey confirmatory factor analysis

Confirmatory factor analysis substantiated the hypothesized four-factor structure of the four survey scales. Although the chi square was statistically significant, χ^2^ (98, *N* = 1807) = 1250.44, *p* < .001, this is due, in part, to the large sample size [24]. All other model fit statistics fell within recommended standards, as defined by Hu and Bentler [23]. The comparative fit index of 0.95 and the root-mean-square error of approximation of 0.08 both suggest an adequate model fit. The standardized root mean square residual of 0.04 was less than 0.08, which is indicative of a good model fit.

### (procedures)

#### Score content

All 6 educators agreed that they could assess student engagement by looking at the data components comprising the engagement score.

#### Score internal structure

Descriptive statistics for the engagement score are presented in Table 2. Using the polychoric correlation matrix of the four components of the engagement score, CFA results confirmed that the composite score was a unidimensional measure. A one-factor solution yielded acceptable model fit. Chi-square is statistically significant (χ^2^_(df)_ = 50.64 _2_, *p* < .0001), in part due to the large sample size. Other model fit indices are within recommended standards [23]. The comparative (CFI) and normed fit (NFI) indices were both 0.95 and the standardized root mean square residual (SMR) was 0.04. The composite reliability index for the latent trait was .70 [25]. All loadings of the engagement score metrics on the latent trait of engagement were positive and statistically significant. Regarding reliability of score generation, the engagement score reflects the student’s actions on that specific case, and as long as the student performed the same actions on that case the computer generated score would be identical. The computer-based scoring underlying the engagement meter thus provides essentially perfect test-retest reliability.

**Table 2:** Descriptive statistics for the components of the engagement score

Variable	Mean score	Standard Deviation
Time	90.6	11.4
MCQ	57.7	9.3
Clinical Reasoning	41.1	34.3
Summary Statement	83.3	28.3

#### Score relationship with other variables

Table 3 presents results from the reliability and correlation analyses. As indicated, all reliability estimates were above recommended guidelines and are considered good [26]. The correlations, although small, were statistically significant and in the predicted directions. In particular, the engagement metric was positively correlated with task value (*r* = .19, *p* < .001), elaboration (*r* = .14, *p* < .001), and engagement (*r* = .17, *p* < .001). On the other hand, the engagement metric was negatively associated with boredom (*r* = −.18, *p* < .001). Finally, all four self-report measures of motivational, emotional, and cognitive engagement were moderately correlated with one another, as would be predicted [2].

**Table 3:** Correlations between the engagement score and several self-report measures of motivational, emotional, and cognitive engagement (*N* = 1807)

	No. of Survey Items	Task value	Boredom	Elaboration	Engagement
Engagement Score	–	.19	−.18	.14	.17
Task value	5	(.93)	−.42	.68	.71
Boredom	4		(.93)	−.28	−.38
Elaboration	4			(.90)	.75
Engagement	3				(.88)

Note: Cronbach’s alphas for the self-report scales are presented in parentheses along the diagonal. All correlations are statistically significant at the *p* < .001 level

#### Score consequences

Mean Pearson correlation for rater agreement with the empirically derived scoring cut-points was 0.98. Intra-class correlations of the ratings of the four faculty members who scored the 10 cases as either low, moderate, or good was calculated to be 0.98, which is considered quite good. Engagement score distributions after implementation of the engagement meter were 86% good, 12% moderate, and 2% low.

## Discussion

There is good evidence for the importance of engagement in online learning, and the present investigation provides initial validity evidence for a machine-generated engagement metric based on student interactions in an online learning environment. The engagement metric appears to provide meaningful insight into students’ learning processes that is not otherwise readily available. We believe the principles used in developing this tool could be generalized to many other forms of online learning.

The engagement score is based on details of use, including time on each page of a case. One motivation for developing the engagement metric was to provide a better indicator of engagement for educators than simply total time to completion of a case. While there is a legitimate argument that we do not know what students are doing during that time, we can reasonably conclude that a very short time (< 20 s) on a page means they are not engaged. Similarly, other components of our engagement score may perform better at identifying low levels of engagement. Answering multiple-choice questions correctly, frequently utilizing the clinical reasoning toolbar, and writing a meaningful summary statement all reflect a higher level of engagement with the case than not doing so. With the scoring cut-points used in the engagement meter, students could not achieve a score of moderate or good by time alone, and a score of good could only be achieved by using the clinical reasoning toolbar frequently, and/or writing a meaningful summary statement. Using a combination of different variables that reflect engagement with the case provides more meaningful data to educators than simply total time spent on the case.

We operationalized engagement as the integration and utilization of students’ motivations and learning strategies in the course of their learning. Pekrun’s control-value theory of achievement emotions [27] views engagement as an indicator of motivation and emotion. Based on this theory, Artino [4] suggested that educators should create learning environments that foster control and value for students, which can thereby improve their chances of positively impacting students’ achievement emotions, as well as their subsequent motivation, learning, and performance. As such, an engagement metric can be a useful indicator of both positive and negative interactions of learners with their environment. Recent work in higher education settings suggests that students who experience negative affect are less likely to use deeper processing strategies, as these require much more engagement and a positive approach to the academic task [28]. In contrast, positive emotions are generally thought to result in greater engagement and the use of deeper processing strategies [29].

The consequences of using the engagement metric must also be considered when evaluating the measure’s validity [30]. Although there is strong conceptual support for the importance of engagement in producing high-quality learning, engagement still cannot (and should not) be considered a direct measure of learning. We thus propose three potential uses of an engagement metric in medical education settings.

### Use an engagement metric to better understand student interactions with the VP to improve the online learning environment

The VPs within which the engagement meter was implemented are focused on learning and not assessment; educators are not given access to data on student performance. This implementation of VPs is intended to create a “safe” learning environment in which students are intrinsically motivated to learn from the VPs. In real world practice, however, students are often required to complete cases [12], and such a requirement may have the effect of shifting students to more extrinsic motivation. This complex interplay of motivational, emotional and cognitive engagement is difficult to tease out. Work by Hege [13] investigated different integration strategies and suggests that voluntary case use with exam relevance of the VPs is preferable to compulsory strategies. However, Kim [12] demonstrated that self-directed use of VPs resulted in rather low levels of use. Educators have legitimate reasons to want to know more about how their students are learning and the engagement meter gives the educator a “peek” into the black box without disrupting the safety of the learning environment by turning the VP into an assessment tool.

The manner in which any online learning is integrated in the curriculum reflects one aspect of the social-cognitive learning environment, and has an effect on student perceptions of their learning [31]. Little is known about the most effective strategies for integrating VPs, but a first principle is that “good” integration strategy will improve, not worsen engagement. Integration strategies can be designed to improve engagement, which can then be easily measured using this approach.

### Use an engagement metric to improve instructional design

We know that high-quality instructional design is crucial to promoting learning [32], but the instructional design features of VPs that promote learning generally, or learner engagement more specifically, are poorly understood. Online learning with instructional design elements that engage the learner are likely to produce better learning outcomes than instructional designs that are not engaging. Directly measuring the learning effectiveness of differing instructional designs is challenging [33], but we believe that education researchers can, appropriately, resort to surrogate measures like the engagement meter. A computer-generated measure of engagement, such as the engagement metric we developed, can be an important tool in refining instructional design. Such information could be actively fed back to the student while learning online [34]. With an embedded engagement metric, design features in VP cases or other forms of online learning can be altered, and the effect on learner engagement can be readily measured.

### Use an engagement metric to better interpret analytics output

Learning analytics offers great potential to understand and improve online learning, and the learning process. Online learning analytics might be aimed at assessing understanding, evaluating knowledge, or predicting other learning outcomes. There are, however, potential pitfalls when trying to add meaning to data analytics, as the accuracy of any of these assessments will be affected by learner engagement. Clearly, poor learning outcomes in an unengaged learner are a different concern than the same learning outcomes in an engaged learner. Understanding student interactions with the learning environment will be critical as we continue to determine the best ways to use learning analytics.

#### Limitations

Our study was based on engagement in virtual patients, and the specific engagement metric developed would need to be altered if used in a different form of online learning. Although we found statistically significant correlations between the engagement metric and the self-report scales, the effects sizes were small, indicating that the majority of the variance in the engagement metric is explained by other factors. This is an important limitation of the present study, but it is not unexpected if one considers the known variability in the learning environment and the fact that these two measurement types are quite different (i.e., a metric based on actions within a case [i.e., learner behavior] vs. a metric based on self-report). To some extent, with the introduction of the engagement meter into VP cases, educators are adding extrinsic motivation to what was once a more purely intrinsically motivated environment. This is an important concern, and one that deserves further investigation.

## Conclusions

A valid measure of student engagement in an online learning environment opens the door to translational research that connects knowledge of the learning sciences to practical educational interventions. A computer-generated engagement metric, which we have demonstrated can be applied across a large number of users and VP cases, can provide some important insights into the learning processes students engage with during online learning. These insights have the potential to drive improvements in other forms of online learning. Clearly, however, these promising results should be corroborated with investigations that explore engagement as a mediating factor in promoting greater learning, and, potentially, as a valuable outcome in and of itself.

## Abbreviations

CFA: Confirmatory factor analysis
MCQ: Multiple choice question
VP: Virtual patient
